# Combined and individual tumor-specific expression of insulin-like growth factor-I receptor, insulin receptor and phospho-insulin-like growth factor-I receptor/insulin receptor in primary breast cancer: Implications for prognosis in different treatment groups

**DOI:** 10.18632/oncotarget.14082

**Published:** 2016-12-21

**Authors:** Sofie Björner, Ann H. Rosendahl, Maria Simonsson, Andrea Markkula, Karin Jirström, Signe Borgquist, Carsten Rose, Christian Ingvar, Helena Jernström

**Affiliations:** ^1^ Department of Clinical Sciences Lund, Lund University Faculty of Medicine, Oncology and Pathology, Lund, Sweden; ^2^ Department of Oncology and Haematology, Skåne University Hospital, Sweden; ^3^ CREATE Health and Department of Immunotechnology, Lund University, Medicon Village, Lund, Sweden; ^4^ Department of Clinical Sciences Lund, Lund University, Skåne University Hospital, Surgery, Lund, Sweden

**Keywords:** insulin-like growth factor-I receptor, insulin receptor, phospho-insulin-like growth factor-I receptor/insulin receptor, breast cancer, prognosis

## Abstract

Clinical trials examining insulin-like growth factor-I receptor (IGF1R)-targeting strategies have emphasized that better predictive biomarkers are required to improve patient selection.

Immunohistochemical tumor-specific protein expression of IGF1R, insulin receptor (InsR), and phosphorylated IGF1R/InsR (pIGF1R/InsR) individually and combined in relation to breast cancer prognosis was evaluated in a population-based cohort of 1,026 primary invasive breast cancer patients without preoperative treatment diagnosed in Sweden. IGF1R (*n* = 923), InsR (*n* = 900), and pIGF1R/InsR (*n* = 904) combined cytoplasmic and membrane staining was dichotomized. IGF1R^strong^/InsR^mod/strong^/pIGF1R/InsR^pos^ tumors were borderline associated with 2-fold risk for events, HR_adj_ (2.00; 95%CI 0.96-4.18). Combined IGF1R and pIGF1R/InsR status only impacted prognosis in patients with InsR^mod/strong^ expressing tumors (*P*_interaction_ = 0.041). IGF1R^strong^ expression impacted endocrine treatment response differently depending on patients’ age and type of endocrine therapy. Phospho-IGF1R/InsR^pos^ was associated with lower risk for events among non-endocrine-treated patients irrespective of ER status, HR_adj_ (0.32; 95%CI 0.16-0.63), but not among endocrine-treated patients (*P*_interaction_ = 0.024). In non-endocrine-treated patients, pIGF1R/InsR^pos^ was associated with lower risk for events after radiotherapy, HR_adj_ (0.31; 95%CI 0.12-0.80), and chemotherapy, HR_adj_ (0.29; 95%CI 0.09-0.99). This study highlights the complexity of IGF hetero-and homodimer signaling network and its interplay with endocrine treatment, suggesting that combinations of involved factors may improve patient selection for IGF1R-targeted therapy.

## INTRODUCTION

The insulin-like growth factor (IGF) axis is involved in many cancer types. However, the prognostic importance of the IGF-receptor family in breast cancer is unclear. The signaling network is complex and involves both the IGF-I receptor (IGF1R) and the insulin receptor (InsR). These receptors can form both homo- and heterodimer (hybrid) receptors [1]. Upon ligand-mediated activation, the receptors become phosphorylated at various tyrosine-kinase residues, which initiate further downstream signal transduction involving the phosphatidylinositol 3-kinase (PI3K) and mitogen activated protein kinase (MAPK) pathways. Furthermore, there is crosstalk between the IGF-signaling network and other signaling pathways e.g. the estrogen receptor (ER) [2–4].

Targeting the IGF signaling pathway was suggested by leading breast cancer experts as a promising approach to find more effective treatment regimens [5]. Several clinical trials with compounds targeting either IGF ligands or receptor have shown promising results in phase I. However, results from phases II and III in unselected patients have not been as encouraging [6]. This could be a consequence of treating unselected groups of patients and a reflection of the complexity of feedback loops and crosstalk with other signaling pathways [3]. It is therefore desirable to find biomarkers that can predict which patients could benefit from IGF-targeting therapies such as small-molecule inhibitors, anti-receptor antibodies, and anti-ligand antibodies [1, 7–11].

The IGF signaling network may also confer resistance to other treatments including HER2-targeted therapy, radiotherapy, endocrine therapy, and chemotherapy [12–15]. The expression patterns of IGF1R, InsR and pIGF1R are therefore important to elucidate in this complex network, its involvement in breast cancer, and its association with prognosis in different treatment groups. There are several reports addressing the prognostic value of these markers individually [16–19]. However, to our knowledge the combination of these three markers regarding prognosis has not been investigated. Given that signaling can occur via both hetero- and homodimers of IGF1R and InsR, the expression of both receptors needs to be investigated. In this study, the expression of IGF1R, InsR and pIGF1R/InsR was evaluated in tumors from 946 primary breast cancer patients in a prospective cohort. We hypothesized that a combination of IGF1R, InsR and pIGF1R/InsR may better identify subgroups of patients with poor outcome than either marker alone, and that tumors with IGF1R^strong^/InsR^mod/strong^/pIGF1R/InsR^pos^ expression are associated with worse prognosis and involved in treatment resistance. The aim was to clarify the importance of these three markers individually and combined regarding risk for breast cancer events, distant metastasis, and overall survival in different treatment groups.

## RESULTS

### IGF1R, InsR and pIGF1R/InsR in relation to tumor and patient characteristics

**Figure 1:**
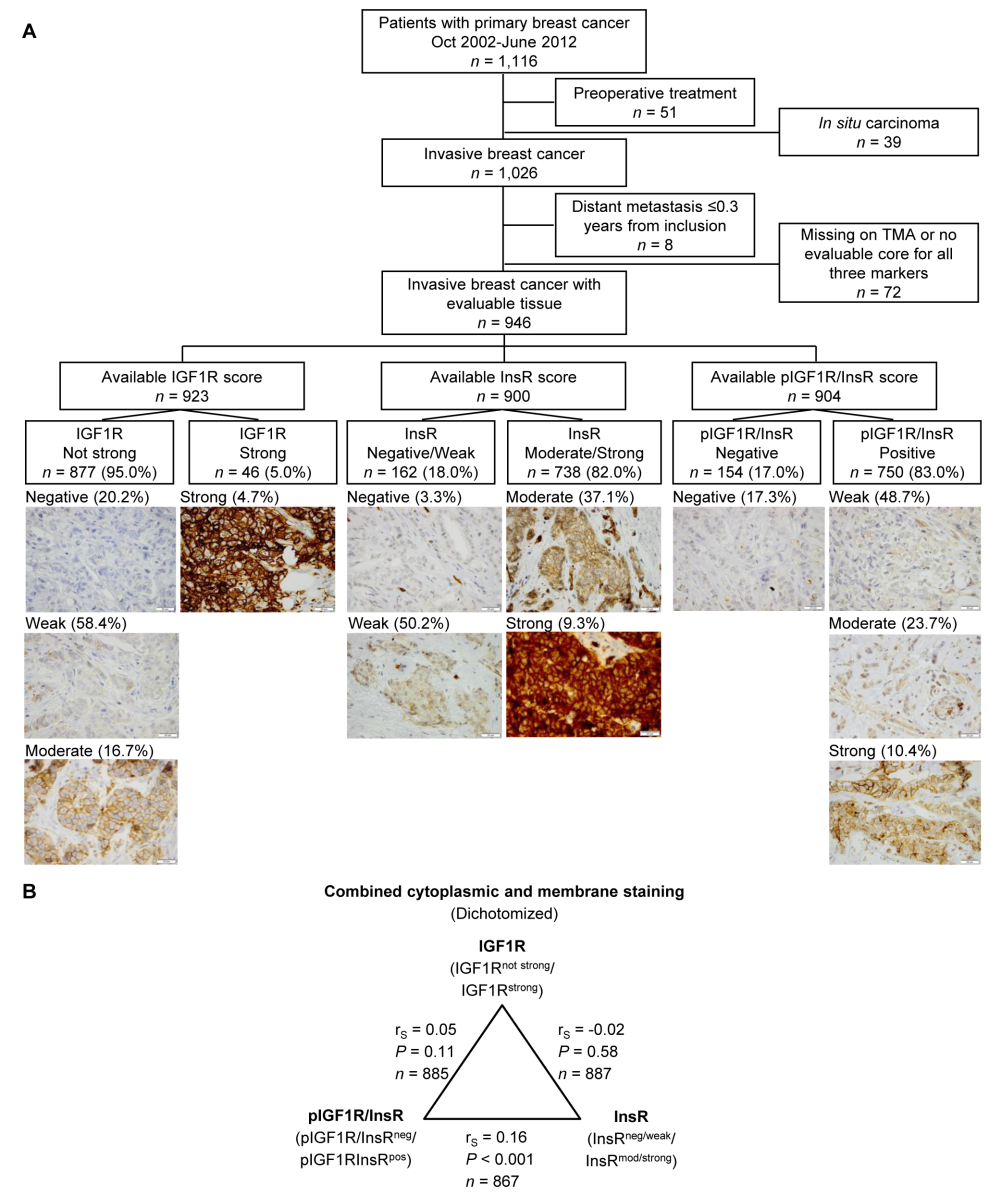
**A**. Flow chart of study population and division of evaluated markers. Total available staining scores for each group are based on combined cytoplasmic and membrane staining. Representative images for IGF1R, InsR and pIGF1R/InsR are shown (scale bar = 20 μm). The corresponding percentages of tumors with different staining intensities indicate cytoplasmic staining only. **B**. Correlations between IGF1R, InsR and pIGF1R/InsR for combined cytoplasmic and membrane staining.

**Figure 2:**
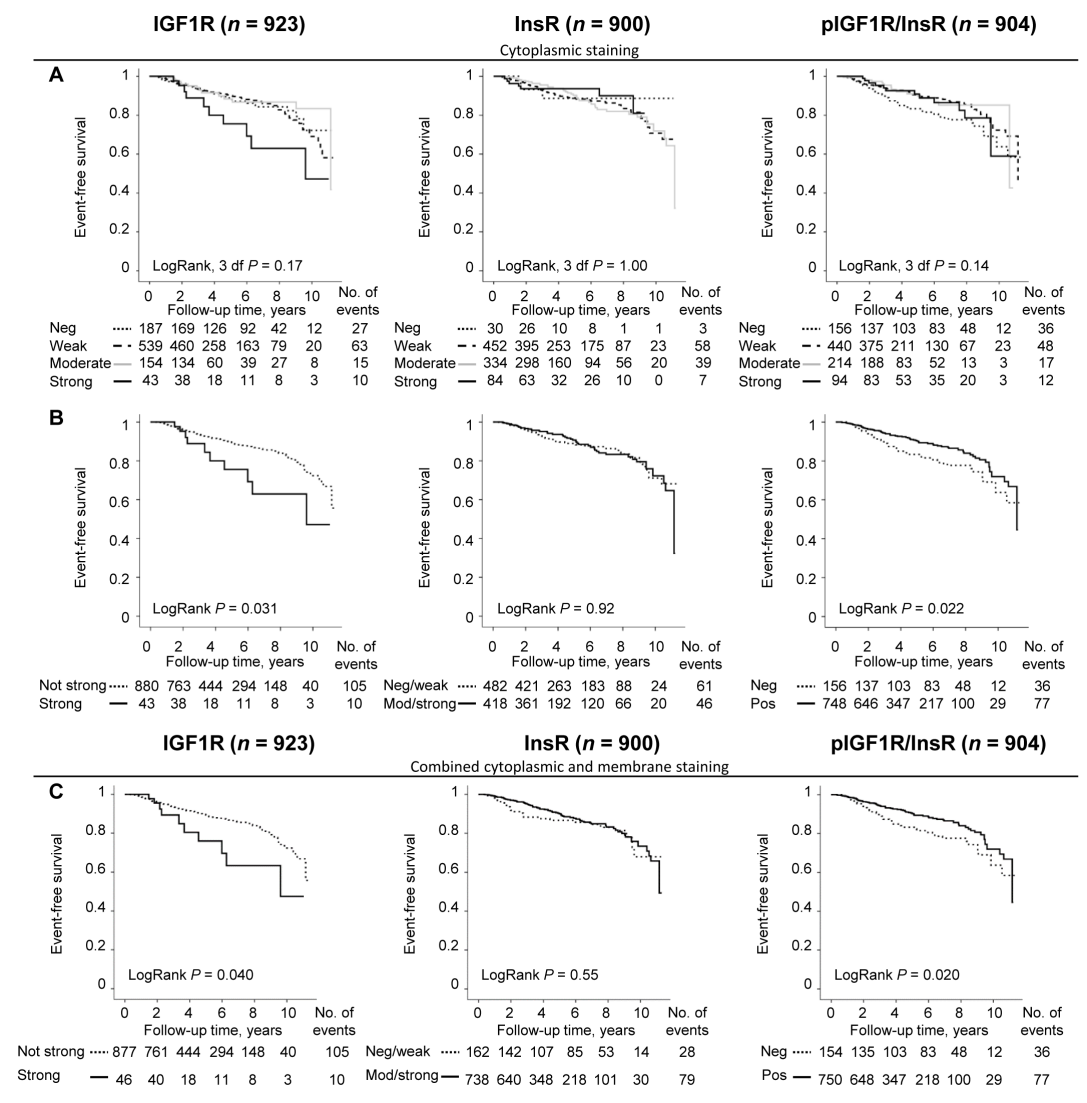
Cut-off determination based on the prognostic significance of IGF1R, InsR and pIGF1R/InsR using Kaplan-Meier plots Curves representing **A**. four different cytoplasmic staining intensities, **B**. dichotomized groups after merging overlapping cytoplasmic staining intensity curves, and **C**. combined cytoplasmic and membrane staining as individual prognostic markers in relation to event-free survival.

The distribution of the staining intensities for IGF1R (*n* = 923), InsR (*n* = 900) and pIGF1R/InsR (*n* = 904) is illustrated in Figure 1A. There were significant correlations between all markers for both cytoplasmic and membrane staining intensities (all *P*-values ≤0.021). For the combined dichotomized cytoplasmic intensity and membrane expression there was only a significant correlation between InsR and pIGF1R/InsR expressions (r_S_ = 0.16, *P*<0.001; Figure 1B). The cut-off determination for prognostically relevant staining intensities for each marker was determined using Kaplan-Meier curves (Figure 2A). Groups where the curves crossed over were combined into one group (Figure 2B). The final dichotomized groups were based on both cytoplasmic and membrane staining (Figure 2C). For IGF1R and pIGF1R/InsR the dichotomized groups remained essentially the same after addition of membrane staining; IGF1R^not strong^ (95.0%) and IGF1R^strong^ (5.0%); pIGF1R/InsR^neg^ (17.0%) and pIGF1R/InsR^pos^ (83.0%). There was no clear separation of tumors stained for InsR and these were therefore grouped into InsR^neg/weak^ and InsR^mod/strong^. The number of patients with InsR^mod/strong^ tumors increased substantially after addition of membrane staining; InsR^neg/weak^ (18.0%) and InsR^mod/strong^ (82.0%).

Associations between the evaluated markers and tumor and patient characteristics are shown in Table 1. IGF1R^strong^ tumor expression was associated with larger invasive tumor size (*P* = 0.001), increasing axillary lymph node involvement (*P* = 0.040), and higher histological grade (*P* = 0.013). IGF1R^strong^ expression was further significantly associated with triple-negative tumors (*P* = 0.014), whereas triple negativity was significantly associated with InsR^neg/weak^ (*P* = 0.021) and pIGF1R/InsR^neg^ (*P* = 0.014) expression. Both InsR^mod/strong^ and pIGF1R/InsR^pos^ expression were associated with ER+ status (*P* = 0.005 and *P* = 0.021, respectively). IGF1R^strong^ expression was associated with a higher frequency of patients receiving any endocrine treatment (*P* = 0.014), and specifically tamoxifen (*P* = 0.029), compared with IGF1R^not strong^ expression. In contrast, a larger percentage of patients with tumors with InsR^neg/weak^ expression had received tamoxifen treatment (*P* = 0.029), compared with patients with tumors with InsR^mod/high^ expression. Height was the only anthropometric factor associated with InsR (*P* = 0.004). None of the other investigated anthropometric factors were associated with any of the other markers.

**Table 1:** Tumor and patient characteristics in relation to tumor-specific expression of IGF1R, InsR and pIGF1R/InsR

	IGF1R		InsR		pIGF1R/InsR	
	Not strong *n* =877 (95%) *n* (%), Median (IQR) or %	Strong *n* =46 (5%) *n* (%), Median (IQR) or %	Negative/Weak *n*=162 (18%) *n* (%), Median (IQR) or %	Moderate/Strong *n*=738 (82%) *n* (%), Median (IQR) or %	Negative *n* =154 (17%) *n* (%), Median (IQR) or %	Positive *n* =750 (83%) *n* (%), Median (IQR) or %
*Tumor characteristics*						
Invasive tumor size						
1 (≤20 mm)	647 (73.8)	24 (52.2)	114 (70.4)	537 (72.8)	119 (77.3)	532 (70.9)
2+ (≥21 mm or skin or muscular involvement independent of size)	230 (26.2)	22 (47.8)	48 (29.6)	201 (27.2)	35 (22.7)	218 (29.1)
Axillary lymph node involvement						
0	537 (61.4)	23 (50.0)	94 (58.0)	453 (61.5)	86 (56.2)	464 (61.9)
1-3	264 (30.2)	15 (32.6)	46 (28.4)	227 (30.8)	47 (30.7)	227 (30.3)
≥4	74 (8.5)	8 (17.4)	22 (13.6)	56 (7.6)	20 (13.1)	58 (7.7)
Missing	2	0	0	2	1	1
Histologic grade						
I	224 (25.6)	6 (13.0)	37 (22.8)	181 (24.6)	39 (25.3)	185 (24.7)
II	436 (49.8)	22 (47.8)	86 (53.1)	366 (49.7)	71 (46.1)	376 (50.2)
III	216 (24.7)	18 (39.1)	39 (24.1)	190 (25.8)	44 (28.6)	188 (25.1)
Hormone receptor status						
ER^+^	775 (88.6)	38 (82.6)	132 (81.5)	658 (89.4)	126 (82.4)	667 (89.1)
PR^+^	630 (72.0)	28 (60.9)	114 (70.4)	523 (71.1)	110 (71.9)	529 (70.6)
Missing	2	0	0	2	1	1
HER2 amplification^a^	67 (11.4)	3 (9.7)	8 (10.5)	57 (10.9)	9 (14.1)	60 (11.0)
Missing	288	15	86	213	90	203
Triple-negative (ER^-^PR^-^HER2^-^)	46 (7.3)	6 (19.4)	12 (14.8)	41 (7.3)	11 (15.1)	40 (6.9)
Missing	243	15	81	174	81	169
*Treatment by last follow-upb*						
Ever chemotherapy	219 (25.0)	15 (32.6)	41 (25.3)	187 (25.3)	34 (22.1)	196 (26.1)
Ever radiotherapy	564 (64.3)	28 (60.9)	103 (63.6)	471 (63.8)	92 (59.7)	485 (64.7)
ER^+^ only						
Ever endocrine therapy	605 (78.0)	36 (94.7)	105 (79.5)	518 (78.6)	100 (79.4)	525 (78.6)
Ever tamoxifen	454 (58.5)	29 (76.3)	90 (68.2)	382 (58.0)	82 (65.1)	392 (58.7)
Ever aromatase inhibitor	300 (38.7)	17 (44.7)	49 (37.1)	259 (39.3)	53 (42.1)	255 (38.2)
*Patient characteristics*						
Age at diagnosis, years	61.1 (52.5-68.0)	60.8 (52.0-69.1)	60.2 (50.2-66.9)	61.4 (53.0-68.2)	61.2 (54.6-69.4)	61.1 (52.3-67.9)
Weight, kg	70.0 (62.0-79.0)	66.5 (58.3-74.0)	68.0 (61.0-76.0)	70.0 (62.0-79.6)	70.0 (62.0-79.0)	70.0 (62.0-78.5)
Missing	22	2	3	20	3	19
Height, m	1.66 (1.62-1.70)	1.64 (1.60-1.68)	1.64 (1.60-1.68)	1.66 (1.62-1.70)	1.65 (1.61-1.69)	1.66 (1.62-1.70)
Missing	22	2	3	20	2	20
BMI, kg/m^2^	25.2 (22.6-28.5)	24.4 (21.8-27.7)	25.0 (22.9-27.7)	25.2 (22.4-28.7)	25.3 (22.7-28.6)	25.1 (22.5-28.3)
Missing	24	2	3	22	3	21
Waist-to-hip ratio, m/m	0.86 (0.81-0.90)	0.87 (0.82-0.90)	0.85 (0.80-0.90)	0.86 (0.81-0.91)	0.84 (0.80-0.90)	0.86 (0.81-0.90)
Missing	31	4	3	29	4	29
Total breast volume ≥850 mL	424 (57.0)	21 (61.8)	83 (61.5)	349 (55.7)	78 (60.0)	359 (56.7)
Missing	133	12	27	111	24	117

Abbreviations: IQR, interquartile range

^a^ HER2 was routinely analyzed in patients <70 years with invasive tumors as of November 2005.

^b^ Patients may have received more than one type of treatment prior to any event.

### The prognostic impact of individual markers

Patients were followed for up to 11 years with a median follow-up of 5.0 years for patients still at risk. Of the 946 patients, 115 had had any breast cancer event of which 72 patients had a distant metastasis and 91 patients died due to any cause. Of the 91 patients who died, 55 patients had had a reported breast cancer event prior to death.

Patients with tumors with IGF1R^strong^ expression was significantly associated with breast cancer events (LogRank *P* = 0.040; Figure 2C). However, IGF1R^strong^ expression was not an independent prognostic marker for any breast cancer event, adjusted HR (HR_adj_) (1.60; 95% CI 0.82-3.07), distant metastasis, or overall survival in multivariable Cox regression analyses.

InsR did not provide any significant prognostic information regarding event-free survival (Figure 2C) or distant metastasis. However, patients with InsR^mod/strong^ expressing tumors had a borderline decreased risk of death due to any cause after adjusting for other prognostic factors and tumor storage time, HR_adj_ (0.66; 95% CI 0.41-1.06).

Patients with pIGF1R/InsR^pos^ tumors had significantly longer event-free survival compared to patients with pIGF1R/InsR^neg^ tumors (LogRank *P* = 0.020; Figure 2C). Multivariable analysis revealed that pIGF1R/InsR did not provide independent prognostic information regarding event-free survival after adjustment for other prognostic factors and tumor storage time, HR_adj_ (0.70; 95% CI 0.46-1.06). Distant metastasis-free survival and overall survival were not associated with pIGF1R/InsR in the multivariable models.

### The prognostic significance of combining IGF1R, InsR and pIGF1R/InsR

**Figure 3:**
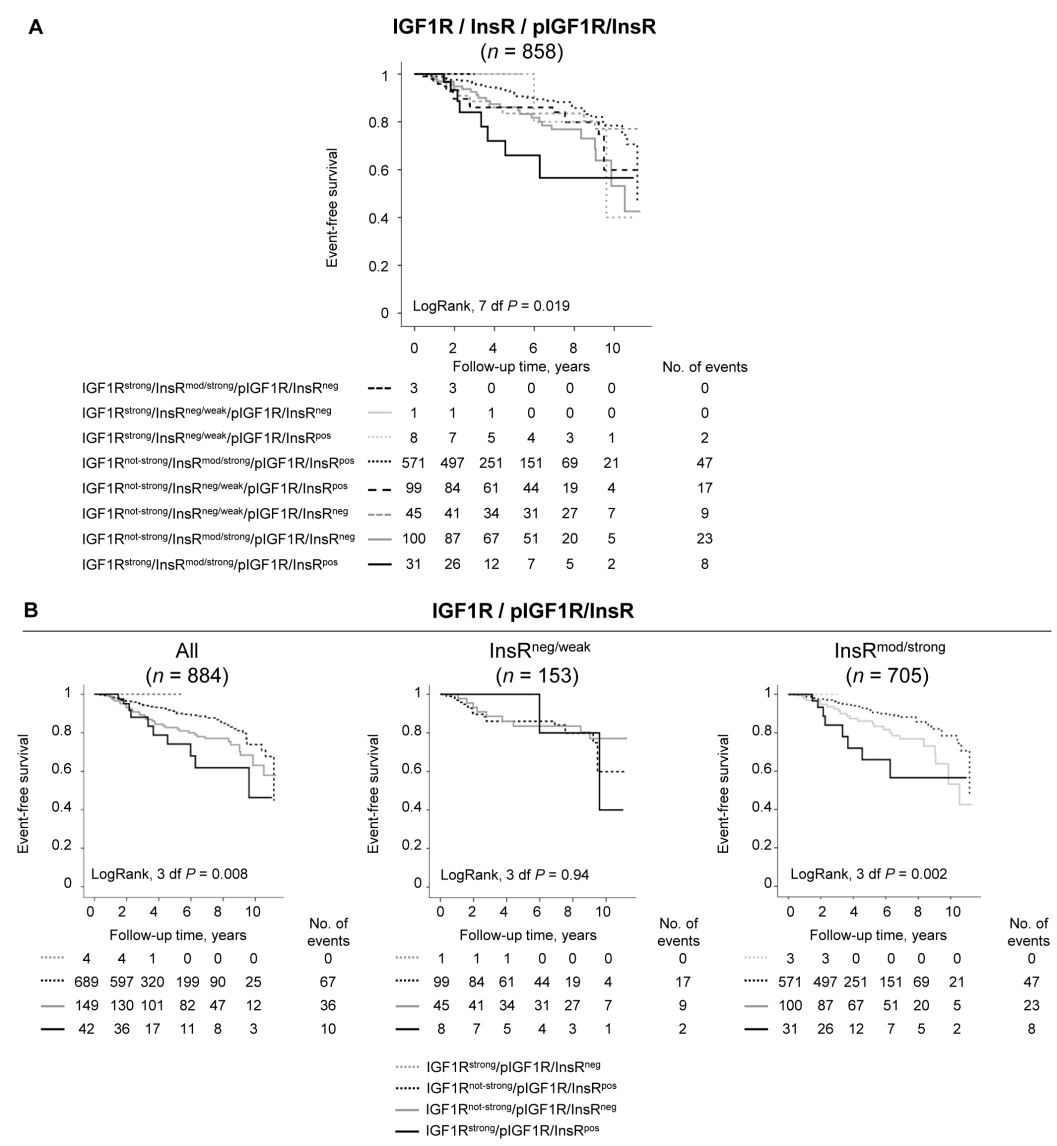
The prognostic value of various IGF1R, InsR and pIGF1R/InsR combinations Kaplan-Meier curves showing event-free survival in relation to **A**. the eight different IGF1R, InsR, and pIGF1R/InsR combinations, and **B**. the four different IGF1R and pIGF1R/InsR combinations among all patients or stratified according to InsR status, (p_interaction_ = 0.041).

The combined expression of IGF1R, InsR, and pIGF1R/InsR was investigated and resulted in eight combinations with different impacts on patient prognosis. In total, 858 tumors were successfully scored for all markers. Patients having IGF1R^strong^/InsR^mod/strong^/pIGF1R/InsR^pos^ tumors had significantly shorter event-free survival compared with all other groups (LogRank 7 df, *P* = 0.019; Figure 3A). A non-significant 2-fold increased risk for breast cancer events was observed among patients with IGF1R^strong^/InsR^mod/strong^/pIGF1R/InsR^pos^ tumors compared with all other groups after adjustment for other prognostic factors and tumor storage time, HR_adj_ (2.00; 95% CI 0.96-4.18).

Since IGF1R and pIGF1R/InsR provided individual prognostic information in the univariable models, these two markers were combined. Patients with tumors with both IGF1R^strong^ and pIGF1R/InsR^pos^ expression had the highest risk for events, followed by tumors with IGF1R^not strong^ and pIGF1R/InsR^neg^ expression, while patients with tumors with IGF1R^not strong^ and pIGF1R/InsR^pos^ expression had a better prognosis (LogRank 3 df, *P* = 0.008; Figure 3B). There were only four patients with IGF1R^strong^ expressing tumors in combination with and pIGF1R/InsR^neg^. While InsR did not provide any individual prognostic information regarding breast cancer events, there was a significant effect modification of InsR on the association between the combined IGF1R and pIGF1R/InsR markers and event-free survival (*P*_interaction_ = 0.041). Further stratification by InsR expression revealed that while no association was seen in InsR^neg/weak^ (LogRank 3 df, *P* = 0.94), InsR^mod/strong^ expression was required to distinguish between the combinations of IGF1R and pIGF1R/InsR on event-free survival (LogRank 3 df, *P* = 0.002; Figure 3B).

### The prognostic impact of IGF1R, InsR and pIGF1R/InsR in different treatment groups

The expression of the three markers in relation to response to endocrine treatment, radiotherapy and chemotherapy was assessed due to the reported influence of IGF-signaling on cancer treatments. Any breast cancer event was used as a marker for poor treatment response.

Neither InsR nor pIGF1R/InsR was associated with outcome among endocrine-treated patients with ER+ tumors, irrespective of type of endocrine treatment. However, there was a borderline significant 2-fold increased risk for recurrence among the patients with ER+ tumors who ever received any type of endocrine treatment and had IGF1R^strong^ tumor expression compared with IGF1R^not strong^ tumor expression, HR_adj_ (2.07; 95% CI 0.98-4.37) adjusted for prognostic markers and treatment with chemotherapy and radiotherapy. Since aromatase inhibitor (AIs) are mainly offered to postmenopausal women, patients were stratified according to age. Among AI- but not tamoxifen (TAM)-treated patients ≥50 years (*n* = 148), IGF1R^strong^ expression was associated with increased risk of breast cancer events, HR_adj_ (4.45; 95% CI 1.12-17.73). This association was not significant in patients ≥50 years who received both AI and TAM switch treatment (*n* = 142), HR_adj_ (2.33; 95% CI 0.42-12.99), and not observed in patients ≥50 years treated with TAM but not AIs (*n* = 246), HR_adj_ (0.80; 95% CI 0.10-6.58). In contrast, among the younger TAM-treated patients <50 years (*n* = 107) with or without AIs, IGF1R^strong^ expression was weakly associated with increased risk of breast cancer events, HR_adj_ (3.91; 95% CI 0.67-22.83). The association appeared to be driven by the group of patients who received TAM but not AIs (*n* = 85), HR_adj_ (14.29; 95% CI 1.82-112.25). There were too few AI-treated patients <50 years to allow for separate analyses. Endocrine treatment response was not associated with any of the other markers individually or combined. Neither IGF1R, InsR nor pIGF1R/InsR added any prognostic information alone or combined among patients treated with radiotherapy (*n* = 604) or chemotherapy (*n* = 239).

### Phospho-IGF1R/InsR adds prognostic information in non-endocrine-treated patients

**Figure 4:**
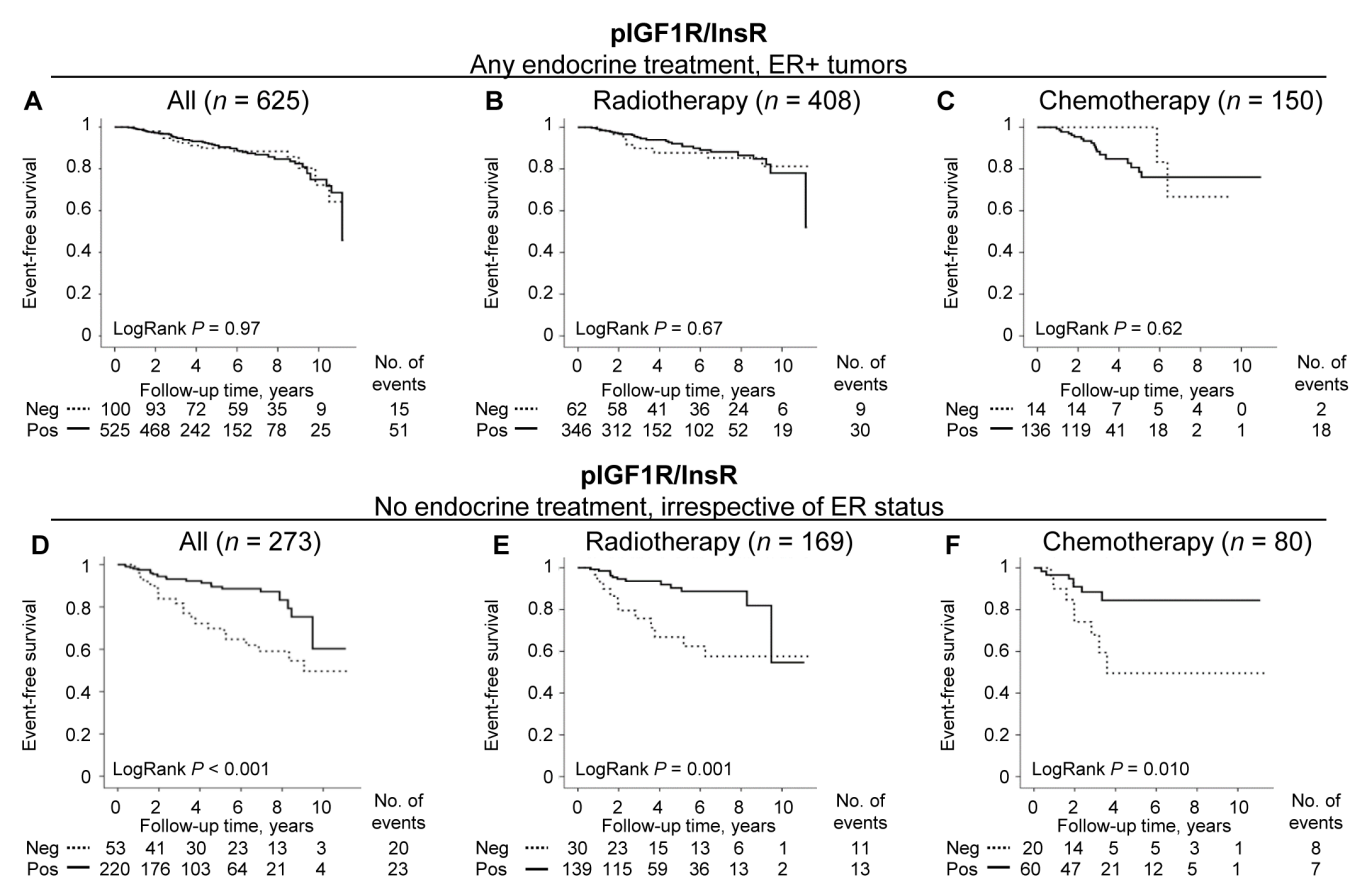
The prognostic importance of pIGF1R/InsR depending on endocrine treatment regarding event-free survival Kaplan-Meier curves presenting **A**.-**C**. endocrine-treated patients with ER+ tumors and **D**.-**F**. non-endocrine-treated patients irrespective of ER status. (B, E) patients also receiving adjuvant radiotherapy, and (C, F) patients also treated with adjuvant chemotherapy. Dashed lines represent patients with pIGF1R/InsR^neg^ tumors, and solid lines pIGF1R/InsR_pos_ tumors.

There was a significant effect modification of any type of endocrine treatment on the prognostic value of pIGF1R/InsR among patients with ER+ tumors (*P*_interaction_ = 0.022). Patients were therefore stratified according to endocrine treatment. As stated above, pIGF1R/InsR was not associated with event-free survival for endocrine-treated patients with ER+ tumors whether or not they had also received radiotherapy or chemotherapy (Figure 4A-4C). Non-endocrine-treated patients with ER+ and pIGF1R/InsR^pos^ tumors had a borderline significant decreased risk of any event after adjustment for prognostic factors and tumor storage time, HR_adj_ (0.39; 95% CI 0.15-1.04). Significant decreased risks for any event with pIGF1R/InsR^pos^ expression were observed among non-endocrine-treated patients with ER- tumors, HR_adj_ (0.27; 95% CI 0.09-0.77), as well as for all non-endocrine-treated patients irrespective of ER status, HR_adj_ (0.32; 95% CI 0.16-0.63; Table 2; Figure 4D). The interaction between pIGF1R/InsR and endocrine treatment was present irrespective of ER status (*Pinteraction* = 0.024). Phospho-IGF1R/InsR was not a prognostic marker for distant metastasis-free survival or overall survival.

**Table 2:** Multivariable Cox regression models for pIGF1R/InsR among non-endocrine-treated patients irrespective of ER status

			Model 1 *n* =248			Model 2^a^ *n* =248			Model 3^b^ *n* =240	
Variables		HR	95% CI	*P*-value	HR	95% CI	*P*-value	HR	95% CI	*P*-value
pIGF1R/InsR	positive	0.32	0.17-0.62	0.001	0.32	0.16-0.63	0.001	0.34	0.17-0.67	0.002
Age	continuous	0.98	0.95-1.01	0.19	0.98	0.95-1.01	0.19	0.98	0.95-1.01	0.17
Invasive tumor size	≥21 mm or skin or muscular involvement	4.90	1.94-12.38	0.001	4.88	1.93-12.35	0.001	4.73	1.89-11.87	0.001
Axillary node involvement	positive	1.77	0.76-4.11	0.19	1.78	0.76-4.18	0.19	1.80	0.77-4.17	0.17
Histological grade	III	1.23	0.41-3.67	0.71	1.21	0.40-3.73	0.74	1.26	0.41-3.84	0.69
ER	positive	0.95	0.29-3.17	0.93	0.93	0.26-3.29	0.91	0.93	0.26-3.29	0.91
BMI	≥25 kg/m^2^	1.10	0.56-2.14	0.79	1.10	0.56-2.14	0.79	1.14	0.58-2.22	0.71

All models are adjusted for treatment; chemotherapy and radiotherapy.

^a^ Adjusted for tumor storage time (years).

^b^ Restriction analysis: Excluding pIGF1R/InsR^pos^ tumors without positive staining for both IGF1R and InsR, or unknown for one or both of the markers (*n*=20 for total and *n*=8 for non-endocrine-treated patients) and adjusted for tumor storage time (years).

23 patients had missing values for one or more adjustment variables.

The prognostic value of pIGF1R/InsR expression was investigated in radiotherapy- (*n* = 169) and chemotherapy- (*n* = 80) non-endocrine-treated patients irrespective of ER status. Radiotherapy-treated patients with pIGF1R/InsR^pos^ tumors had a lower risk for breast cancer events, HR_adj_ (0.31; 95% CI 0.12-0.80; Figure 4E), but not for distant metastasis-free survival or overall survival. A similar association was also seen in chemotherapy-treated patients with pIGF1R/InsR^pos^ tumors, HR_adj_ (0.29; 95% CI 0.09-0.99; Figure 4F). Among the patients treated with chemotherapy, pIGF1R/InsR^pos^ was additionally borderline associated with better prognosis for distant metastasis-free survival, HR_adj_ (0.23; 95% CI 0.05-1.03), and overall survival, HR_adj_ (0.30; 95% CI 0.08-1.08).

The pIGF1R/InsR antibody may cross-react with other activated tyrosine kinase receptors according to the manufacturer. There were only 7 out of 748 (0.9%) pIGF1R/InsR^pos^ tumors with negative staining for both IGF1R and InsR, and additional 13 (1.7%) tumors with expression unknown for one or both of the markers. The effect estimates remained essentially the same after removing these cases in a restriction analysis (Table 2).

### Phospho-IGF1R/InsR adds opposing overall survival information depending on InsR status among non-endocrine-treated patients

The potential involvement of InsR on patient outcome in combination with pIGF1R/InsR was investigated in non-endocrine-treated patients. For patients with tumors with InsR^mod/strong^ expression, pIGF1R/InsR^pos^ was associated with significantly lower risk for events, HR_adj_ (0.26; 95% CI 0.12-0.58), and distant metastasis, HR_adj_ (0.22; 95% CI 0.06-0.85) compared with pIGF1R/InsR negativity. There was no significant interaction between InsR and pIGF1R/InsR when investigating any type of first event or distant metastasis-free survival. However, for overall survival the interaction between the markers indicated a significant effect modification of pIGF1R/InsR depending on InsR status (*P*_interaction_ = 0.030). Phospho-IGF1R/InsR positivity was non-significantly associated with shorter overall survival in patients with InsR^neg/weak^ tumors, but was borderline associated with longer overall survival in patients with InsR^mod/strong^ expression, HR_adj_ (0.32; 95% CI 0.10-1.01).

### Sensitivity analyses

Since there were 15 patients with bilateral tumors, sensitivity analyses were performed. For each of the markers, five patients had no evaluable core on the bilateral side. For the patients with two evaluable tumors, few tumors changed dichotomized status; IGF1R (*n* = 1), InsR (*n* = 3), and pIGF1R/InsR (*n* = 4). The results remained essentially the same in the sensitivity analyses using the dichotomized status for the contralateral tumor in all but two analyses. For InsR^mod/high^ in all patients, the results became significant HR_adj_ 0.60 (95%CI 0.37-0.97), while for IGF1R^strong^ the subgroup of AI-treated patients >50 years old with ER+ tumors, the results became non-significant when using the IGF1R status for the contralateral tumor.

## DISCUSSION

The IGF system has an established mitogenic role in cancer. However, recent clinical trials examining IGF1R targeting therapies have been disappointing and have highlighted the need for improved selection of patients likely to benefit from these treatment strategies. This study demonstrated that IGF1R^strong^ expression was associated with poorer prognosis and impacted endocrine treatment response differently depending on patients’ age and type of endocrine therapy. Phospho-IGF1R/InsR^pos^ expression was independently associated with longer event-free survival, but this association was only observed among non-endocrine treated patients irrespective of ER status. InsR conferred a significant effect modification on the associations between prognosis and combinations of IGF1R and pIGF1R/InsR, where pIGF1R/InsR, IGF1R^strong^/InsR^mod/strong^/pIGF1R/InsR^pos^ tumors were associated with the worst prognosis.

The prognostic importance of tumor IGF1R levels has previously been studied with conflicting results, possibly related to different patient cohort compositions, antibodies, and cut-offs [16, 17, 20, 21]. In the present study, the prognostic relevant cut-offs were determined through exploratory analyses. Groups where the curves crossed over were combined into one group in order to avoid violation of the assumption of proportional hazards. A recent meta-analysis of ten independent studies evaluating cytoplasmic and membrane staining of IGF1R in breast cancer patients concluded that IGF1R was associated with better outcome in hormone receptor positive cancers, but with poor survival in triple-negative breast cancers [20]. This is in contrast to our finding of worse prognosis among patients with IGF1R^strong^ expressing tumors; however IGF1R was not an independent prognostic marker. In the present study, there was a significant association between IGF1R^strong^ expression and triple-negative tumors, larger invasive tumors and axillary lymph node involvement, which are all features of aggressive breast cancer. It may appear contradictory with a significant association between IGF1R^strong^ expression and higher frequency of endocrine treatment, as well as a higher proportion of triple-negative tumors among IGF1R^strong^ tumors compared to the IGF1R^not strong^ tumors. However, the almost all of the patients with ER+ IGF1R^strong^ tumors received endocrine treatment, which may reflect more aggressive tumor features, compared to a significantly lower proportion of endocrine-treated patients with ER+ IGF1R^not strong^ tumors. Given the established mitogenic role of IGF1R from numerous preclinical studies, it is possible that tumors with IGF1R^strong^ expression circumvent regulatory feedback loops and acquire more aggressive characteristics.

Despite a significant association between IGF1R^strong^ expression and higher frequency of endocrine treatment, no association between IGF1R^strong^ expression and ER positivity was detected in this patient cohort. IGF1R^strong^ expression indicated poor prognosis among endocrine-treated patients with ER+ tumors, also after adjustment for chemotherapy and radiotherapy, although this finding should be interpreted with caution due to limited number of patients with tumors with IGF1R^strong^ expression. Further, in one of the subgroups, the results did not remain significant in the sensitivity analyses when the contralateral tumor was used. These results are in line with a previous report showing that IGF1R overexpression is associated with endocrine resistance caused by increased downstream signaling [22], The treatment-predictive role of IGF1R^strong^ expression differed depending on type of endocrine treatment and age of the patient. The shorter event-free survival among endocrine-treated patients with IGF1R^strong^ expressing tumors may reflect general aggressive features related to those tumors. Endocrine-treated patients with IGF1R^strong^ expressing tumors may therefore benefit from additional treatment, possibly an IGF1R-targeting therapy.

In contrast to the expected mitogenic role of IGF1R signaling, pIGF1R/InsR (Tyr1131/Tyr1146) positivity was independently associated with significantly longer event-free survival, consistent with some [18, 23], but not all previous research [19]. However, the association between longer event-free survival and pIGF1R/InsR positivity was only present in non-endocrine-treated patients irrespective of ER status. Although this is not a randomized controlled trial population and endocrine-treated and non-endocrine-treated patients differed in many aspects, we hypothesize that endocrine treatment may be more beneficial for patients with pIGF1R/InsR^neg^ tumors. Exploratory analyses in the present cohort among patients with ER+ and pIGF1R/InsR^neg^ tumors showed a lower risk for breast cancer events for endocrine-treated patients compared to non-endocrine treated patients. In contrast, no difference between endocrine-treated and non-endocrine-treated patients was observed among patients with ER+ and pIGF1R/InsR^pos^ tumors (data not shown). In order to accurately test this hypothesis, the endocrine-treatment predictive value of pIGF1R/InsR should be tested in a randomized controlled trial, such as SBII:2, with patients randomized to TAM or no TAM treatment irrespective of ER status [24].

This study showed that non-endocrine-treated patients with pIGF1R/InsR^pos^ tumors were less likely to have recurrence after radiotherapy and chemotherapy, compared with patients having pIGF1R/InsR^neg^ tumors. The same was not seen in endocrine-treated patients. Tumors with higher proliferation rates are more sensitive to radiotherapy and chemotherapy. Since pIGF1R/InsR positivity indicates active IGF1R-signaling, which promotes cell proliferation, a plausible biological explanation would be that pIGF1R/InsR^pos^ tumors with higher proliferative signals are more responsive to radio- and chemotherapy, than pIGF1R/InsR^neg^ tumors. However, the Ki67 proliferative index was only available as of March 2009 and therefore not further investigated in this study. In support of our findings, it has been shown in experimental models that blocking IGF1R after chemotherapy resulted in better therapeutic response than IGF1R blockade followed by chemotherapy [25]. In contrast to the report by Turner *et al*., IGF1R was not a prognostic marker for radiotherapy response [13].

InsR^mod/strong^ expression was associated with longer overall survival, and both InsR^mod/strong^ and pIGF1R/InsR^pos^ expression was associated with ER+, which confirm the engagement of the two signaling systems [2]. Previously, InsR positivity has been associated with worse outcome [19, 26]. However, earlier studies have focused on individual markers. In the present study, InsR status was important in combination with the other markers. Patients with tumors with both pIGF1R/InsR^pos^ and IGF1R^strong^ expression had a worse prognosis only when the tumor also expressed mod/strong levels of InsR, suggesting formation of IGF1R/InsR heterodimers. This is in line with a previous report demonstratinga direct association between InsR overexpression and increasedabundance of hybrid IGF1R/InsR receptors, relative to IGF1R homodimers, in breast cancer specimens. Furthermore, hybrid receptors were more responsive to IGF-I stimulation with receptor phosphorylation exceeding that of IGF1R [27]. This illustrates the complexity of the IGF1R signaling network and the importance of evaluating several biomarkers in the pathway.

This population-based patient cohort is representative for the southern region of Sweden [28] with a high follow-up rate [29, 30]. Accordingly, the included patients were not randomized to treatment, often received more than one treatment, and may have switched between TAM and AIs. In addition, treatment regimens and guidelines changed over time, which made it challenging to evaluate any treatment specific effects. However, this study includes consecutive primary breast cancer patients and thereby reflects the authentic clinical situation. The missing 72 cases did not differ significantly from the included 946 patients regarding tumor and patient characteristics (data not shown). This indicates that there was no bias between included patients with evaluable tumors and excluded patients with non-evaluable tumors. Each marker was evaluated on a separate tissue section of the tumor core, which enables co-expression of the markers in the same tumor, but not in individual cells. Triple-staining immunohistochemistry could increase the accuracy of the co-staining but is technically challenging due to the risk of cross reactions between antibodies as well as difficulties in distinguishing between three colors. This study also provides support for the notion that staining of some, but not all markers change with storage time and adjustment for storage time may be warranted.

The selected pIGF1R/InsR antibody was chosen based on a previous publication evaluating the expression of pIGF1R/InsR in breast cancer tissue with IHC, and the specificity by treating DU145 prostate cancer cells with a IGF1R tyrosine kinase inhibitor [19]. Since the antibody may cross-react with other tyrosine kinase receptors, restriction analyses were performed in the present study. The results remained essentially the same after removing the few cases that were pIGF1R/InsR^pos^ but negative for both IGF1R and InsR or had tumors where expression for either IGF1R and/or InsR was unknown (*n* = 20). The findings from this study suggest that this pIGF1R/InsR antibody may be a clinically relevant marker since the results obtained using this antibody indicated a strong independent association with prognosis among non-endocrine-treated breast cancer patients. There are two isoforms of the insulin receptor, InsR-A and InsR-B, where InsR-A is the predominant isoform in breast cancer [31]. The selected InsR antibody detects the β subunit, which is identical for the two isoforms and cannot distinguish between them. InsR expression in the present study thus reflects the total amount of InsR that may form heterodimers with IGF1R.

The disappointing results from clinical trials with IGF1R inhibitors may be due to an inadequate selection of patients and emphasize the importance of finding predictive biomarkers. These should also include InsR since it can form heterodimers with IGF1R. Although beyond the scope of this study, aberrant systemic or local IGF ligand levels may additionally predict patients with IGF-dependent tumors likely to respond to IGF-intervention strategies. Body constitution such as weight and BMI may influence IGF-I, insulin and C-peptide levels [32, 33]. In line with this, there are indications that BMI might be important to consider when identifying patients likely to benefit from IGF-targeted therapies [1]. Compared to North American cohorts, the present study population had a relatively low BMI that may influence the relationship between IGF1R signaling and breast cancer prognosis. Data regarding body measurements were available in the present patient cohort, and all multivariable models were adjusted for BMI. The only association observed between body constitution and tumor markers was between height and InsR expression.

Taken together, the data from this study supports the hypothesis that patients with IGF1R^strong^/InsR^mod/strong^/pIGF1R/InsR^pos^ tumors had shorter event-free survival compared to all other groups. InsR significantly modified the impact of the combination of IGF1R and pIGF1R/InsR on prognosis, which may reflect the balance of IGF1R/InsR hetero- and homodimers and thereby receptor activity. IGF1R^strong^ expression was a predictive indicator of age-related endocrine treatment response, and pIGF1R/InsR positivity indicated better response to radiotherapy and/or chemotherapy in non-endocrine-treated patients, irrespective of ER status. This study highlights the complexity of the IGF system including its interplay with endocrine treatment in relation to breast cancer outcome. The results suggest that evaluation of all three markers confer a more comprehensive understanding than individual markers and may lead to improved selection of patients for IGF-treatment regimens.

## MATERIALS AND METHODS

### Patients

The patient material for this study is part of an ongoing cohort, BC Blood Study, and described in detail by Simonsson *et al*. [29]. 1,116 patients diagnosed with primary breast cancer between October 2002 and June 2012 at the Skåne University Hospital, Lund, Sweden, were included. The patients were between 24 and 99 years old at inclusion without other history of cancer within the last ten years. The patients’ body measurements, including weight, height, waist and hip circumference, and breast volume [34, 35], were measured by a research nurse prior to surgery. Patients were additionally asked to fill out questionnaires regarding reproductive history, use of exogenous hormones or other medications, and lifestyle factors including smoking, alcohol and coffee consumption. Clinicopathological information regarding tumor size, lymph node status, histological grade, HER2 (HER2 was routinely analyzed in patients <70 years with invasive tumors as of November 2005; n_missing_ = 263), ER, and progesterone receptor (PR) status were obtained from medical records and pathology reports. Information about breast cancer recurrence or death was collected from patient charts, pathology reports, the Regional Tumor Registry or the Population Registry. Treatment was administered according to standard of care and was recorded until the first breast cancer event. In patients without breast cancer events, treatments were recorded until last follow-up or death prior to July 1, 2014. Written informed consents were obtained from all participating patients and the study was approved by the local ethics committee at Lund University (Dnr 75-02, Dnr 37-08, Dnr 658-09, Dnr 58-12, Dnr 379-12, Dnr 227-13, Dnr 277-15, and Dnr 458-15).

The final study cohort consisted of 946 patients after excluding patients who received preoperative treatment (*n* = 51), patients with only ductal carcinoma *in situ* (*n* = 39), patients with distant metastasis ≤0.3 years from baseline (*n* = 8), and tumors missing from the TMA or no evaluable cores for all three markers (*n* = 72; Figure 1A). Inability to assess staining was due to loss of tumor cores or cores not containing invasive tumor cells. The report followed the REMARK criteria [36].

### Tissue microarray and immunohistochemistry

Dual cores (1.0 mm) from representative tumor regions of formalin-fixed paraffin-embedded (FFPE) tissue blocks were collected from surgical specimens and assembled in a tissue microarray (TMA), using a semi-automated tissue array device (Beeches instruments, Sun Prairie, WI). The FFPE blocks were stored in room temperature prior to sectioning and the 4 μm thick TMA sections were kept in -20°C until immunohistochemical staining. The sections were automatically deparaffinized followed by pretreatment using the PT Link system (DAKO, Glostrup, Denmark). Sections for all three markers were obtained from the same tumor core and sectioned at the same occasion. Immunohistochemistry was subsequently performed for each marker on individual slides using the Autostainer Plus from DAKO with the EnVision FLEX high-pH kit, according to the manufacturer's instructions (DAKO, Glostrup, Denmark). The following antibodies were used: IGF1Rβ (sc-713, Santa Cruz Biotechnology; dilution 1:150), phospho-IGF1Rβ (Tyr1131)/Insulin Receptor β (Tyr1146) (#3021, Cell Signaling Technology; dilution 1:50), Insulin Receptor (β-subunit) (GR36, Calbiochem; dilution 1:50).

Evaluation of the immunohistochemical staining was performed by two independent observers (SBj, AR), without knowledge of tumor characteristics and patient information. In case of discrepancy for any of the three assessed markers and any parameter (5.6%), a re-examination was done until consensus was reached. Scoring included cytoplasmic staining intensity score; negative (neg), weak, moderate (mod), strong, and membrane staining; neg, weak, strong. Since both cytoplasmic and membranous receptors may be of importance, a combined score of cytoplasmic and membrane staining was applied for all three markers. For IGF1R: tumors showing neg, weak or moderate cytoplasmic and/or membrane negative or weak staining were considered IGF1R^not strong^, and the tumors with either strong cytoplasmic or strong membrane staining were classified as IGF1R^strong^. For InsR: tumors with no or weak cytoplasmic staining and no membrane staining were considered InsR^neg/weak^, while moderate and strong cytoplasmic staining and positive membrane staining was considered InsR^mod/strong^. For pIGF1R/InsR: tumors with no staining in the cytoplasm and membrane were considered pIGF1R/InsR^neg^, and those with weak, moderate or strong cytoplasmic and/or membrane staining were considered pIGF1R/InsR^pos^. In case of bilateral tumors (*n* = 15), the highest value was applied. All dichotomized values for cytoplasmic intensity and membrane staining came from the same tumor for each individual marker. Dichotomized scores for the three different markers came from the same tumor except in one case. This patient was therefore removed from all analyses with combined markers (*n* = 858). The tumor characteristics from the same tumor were used in all analyses where tumor characteristics were included. Sensitivity analyses were performed using the values for the bilateral tumor.

### Statistical methods

The SPSS software versions 19 and 22 (IBM) was utilized for all statistical analyses. The following variables were used as continuous variables: age (years), BMI (kg/m^2^), waist-to-hip ratio, height (m), and weight (kg). The subsequent variables were used as dichotomous variables: breast volume (≥850 mL; yes/no), patients who had received postoperative chemotherapy (yes/no), radiotherapy (yes/no), and endocrine therapy (yes/no); the endocrine therapy group was stratified according to TAM treatment (yes/no) and AI treatment (yes/no) prior to any breast cancer event, last follow-up or death. Tumor characteristics included invasive pathologic tumor size (≤20 mm, ≥21-50 mm, ≥51 mm or skin or muscular involvement), axillary lymph node involvement (0, 1-3, 4+), histologic grade (I-III), hormone receptor status (ER^+^, PR^+^), HER2 amplification, and triple-negativity for ER, PR and HER2. Due to few patients with tumors ≥51 mm (*n* = 14) or skin or muscular involvement (*n* = 2) these groups were combined with patients with tumors ≥21 mm for all analyses.

Linear-by-linear association test was used for associations between expression levels and categorical variables. The Mann-Whitney U-test was used to compare medians for continuous variables due to non-normal distributions. The LogRank test was used for univariable survival analyses, and illustrated with Kaplan-Meier curves. Cox regression was used for multivariable analyses providing hazard ratios (HRs) with 95% confidence intervals (CI) adjusted for age (continuous), invasive tumor size (≥21 mm or skin or muscular involvement), any axillary lymph node involvement, histological grade III, ER status, and BMI (≥25 kg/m^2^). Staining intensity may vary depending on storage time for the tumors. Tumor storage time (years) between surgery and staining of IGF1R, InsR and pIGF1R/InsR was therefore investigated with the Mann-Whitney U-test. A significant association between longer tumor storage time and weaker staining intensity was found for InsR (*P*<0.001) and for pIGF1R/InsR (*P*<0.001), while staining intensity did not significantly differ with storage time for IGF1R (*P* = 0.28). Tumor storage time was therefore added to the adjustment variables for analyses of InsR and pIGF1R/InsR. Interaction terms between the combination of IGF1R and pIGF1R/InsR and InsR, and between pIGF1R/InsR and any endocrine treatment, and pIGF1R/InsR and InsR were calculated and used in Cox regression analyses to investigate potential effect modifications prior to stratification.

Patients were followed from inclusion to the first breast cancer event or distant metastasis, respectively, and patients without events were censored at the last follow-up or death prior to July 1^st^ 2014. Breast cancer events were defined as local or regional recurrences, contralateral cancer or distant metastasis. For distant metastasis-free survival, only distant metastases were considered events. Death due to any cause was the only considered event for overall survival.

Power calculations assuming 900 patients with an accrual interval of 10 years and additional follow-up time of two years and a frequency of 5% or 16.7% of IGF1R^strong^, InsR^mod/strong^ and pIGF1R/InsR^pos^ tumors showed that the study was able to detect true HRs between 0.591 and 1.866 for 5%, and 0.731 and 1.412 for 16.7% with 80% power and α of 5% [37].

Two-tailed nominal *P*-values not adjusted for multiple testing are presented. A *P*-value of less than 0.05 was considered significant.

## Abbreviations

IGF1R: insulin-like growth factor receptor 1
InsR: insulin receptor
ER: estrogen receptor
PgR: progesterone receptor
HER2: human epidermal growth factor receptor
TAM: tamoxifen
AI: aromatase inhibitor
PI3K: phosphatidylinositol 3-kinase
MAPK: mitogen activated protein kinase
